# Microstructure and Optical Properties of Nanostructural Thin Films Fabricated through Oxidation of Au–Sn Intermetallic Compounds

**DOI:** 10.3390/ma14144034

**Published:** 2021-07-19

**Authors:** Lukasz Skowronski, Marek Trzcinski, Aleksandra Olszewska, Robert Szczesny

**Affiliations:** 1 Institute of Mathematics and Physics, UTP University of Science and Technology, Kaliskiego 7, 85-796 Bydgoszcz, Poland; marek.trzcinski@utp.edu.pl (M.T.); aleksandra.olszewska@utp.edu.pl (A.O.); 2 Faculty of Chemistry, Nicolaus Copernicus University in Torun, Gagarina 7, 87-100 Torun, Poland; roszcz@umk.pl

**Keywords:** intermetallic compounds, microstructure, Au–Sn, core–shell, thin films

## Abstract

AuSn and AuSn_2_ thin films (5 nm) were used as precursors during the formation of semiconducting metal oxide nanostructures on a silicon substrate. The nanoparticles were produced in the processes of annealing and oxidation of gold–tin intermetallic compounds under ultra-high vacuum conditions. The formation process and morphology of a mixture of SnO_2_ and Au@SnO_*x*_ (the core–shell structure) nanoparticles or Au nanocrystalites were carefully examined by means of spectroscopic ellipsometry (SE), X-ray photoelectron spectroscopy (XPS), scanning electron microscopy (SEM), and transmission electron microscopy (TEM) combined with energy-dispersive X-ray spectroscopy (EDX). The annealing and oxidation of the thin film of the AuSn intermetallic compound led to the formation of uniformly distributed structures with a size of ∼20–30 nm. All of the synthesized nanoparticles exhibited a strong absorption band at 520–530 nm, which is typical for pure metallic or metal oxide systems.

## 1. Introduction

Semiconducting metal oxide nanostructures (SMONs) with a large specific surface area have many active sites that allow them to work as effective catalysts [1,2], active materials in gas-sensing devices [3,4], electrode materials for lithium ion batteries [5], or dye-sensitized solar cells [6]. SMONs based on the transition metal oxides, such as CuO, SnO${}_{2}$, ZnO, TiO${}_{2}$, Fe${}_{2}$O${}_{3}$, In${}_{2}$O${}_{3}$, Co${}_{3}$O${}_{4}$, and WO${}_{3}$, in the form of nanorods, nanoparticles, nanowires, nanospheres, nanosheets, nanotubes, and mesoporous nanostructures [3,7,8,9] are used especially in gas-sensing applications. One method that has been adopted to improve their sensing performance is the incorporation of metal ions [10,11] (such as noble-metal atoms [12,13]) into the oxide structure or the fabrication of multi-phase SMONs [14,15]. SMONs have been demonstrated to provide desirable performance enhancements related to sensitivity, response time, selectivity, reversibility, reproducibility, and long-term stability [3]. Despite the fact that new sensing systems are being developed, there are still some challenges related to the above-mentioned areas of the sensing process. The challenges in this field are, among others, developing sensors that are operated at room temperature (RT) to minimize energy consumption and cost, increasing their sensitivity and stability, and enabling the miniaturization of devices to make them suitable for handheld operations [3].

Simultaneously, the extensive surfaces of oxides (especially heteroatom-doped oxides) are great platforms for catalytic reactions because most of them depend strongly on the structure of the surfaces and interfaces [1,16,17]. Therefore, materials with porous architectures [3] or hierarchical nanostructures [18] and with large specific surface areas can substantially increase their surface activity sites to create excellent catalytic materials for environmental and energy applications [17,19]. In the literature, different kinds of structures were reported: molecular sieves [20], hollow nanocages or spheres [21,22], yolk–shell systems [23,24], and hierarchical structures composed of well-mannered low-dimensional sub-units [25]. Unfortunately, in many cases, various practical factors, e.g., the necessity of using templates, significantly complicate the process of fabrication of desirable materials [1]. The template method is usually coupled with a high-temperature calcination that not only complicates the synthetic route, but may also adversely affect the functionality of the synthesized system.

For both of the above-mentioned applications of the nanostructural oxide-based materials, especially for multi-phase composite systems (e.g., oxide/oxide, metal/oxide), there is still the essential, indispensable challenge of developing facile approaches for the design and fabrication strategies for these kinds of materials [26]. This concerns the structures of SMONs in the form of both powders and nanostructured thin films. The need for a new synthetic strategy is obvious if we consider the use of oxide-supported gold nanoparticles [26] to produce SMONs in the form of an oxide with embedded noble-metal nanocrystallites [26] in contrast to oxide structures with Au-NPs that are deposited after oxide formation. Tuning the parameters that determine the overall performance (conversion and selectivity) of the catalyst, such as the size or dispersion of nanoparticles, the structure and properties of oxide supports, and the gold–oxide interface interactions is essential [17].

Oxide structures with large specific surface areas (in the forms of nanoparticles, nanorods, nanowires, nanoflowers, nanosheets, nanofilms, nanotubes, porous structures, and hierarchical nanostructures) are produced by means of a set of wet chemical methods (e.g., hydrothermal treatment, sol–gel synthesis, precipitation methods, or nanocasting) [3,27,28,29,30,31,32,33]. Solution-based synthesis is a very common, effective pathway for preparing various low-dimensional oxide structures.

Gold nanoparticles (Au-NPs) exhibit desirable properties in the field of catalysis [33,34,35,36]. They can be used in many areas, such as low-temperature CO oxidation [37], nitro reduction [38], and oxidation of hydrocarbons [26]. Au-supported nanoparticles are also most often synthesized by using wet chemical methods: co-precipitation (CP) and deposition–precipitation (DP) [39]. Although the utilization of such methods seems to be a simple and cheap approach to catalysis, the uncontrolled aggregation, poor control of the particle size, and chloride contamination (precursor: HAuCl${}_{4}$) not only prevent the application of these materials, but can also cause the deactivation of the Au catalysts [40]. These inconveniences hinder the use of Au-NPs for many applications. Effective gold-based catalysts can be produced in the form of a metal–oxide core–shell system [33,35]. In contrast to the above-mentioned wet methods, we propose the use of an alternative method (based on the oxidation of thin films of intermetallic compounds (IMCs)) for the fabrication of Au–oxide SMONs, which can overcome these inconveniences.

It is well known that intermetallic compounds exhibit interesting electrical, optical, magnetic, and catalytic properties [41,42,43,44,45,46,47,48,49]. The growing interest in these compounds is caused, among other reasons, by their better high-temperature [50,51] or cycling (in lithium ion batteries) [25,52] stability compared with that of separate metals. Moreover, in contrast to alloys, IMCs provide well-defined stoichiometry, atomic arrangements, and unique electronic and geometric structures, and can therefore be used as low-cost catalysts with tailored performance [5]. The advanced properties of IMCs are directly associated with the newly formed chemical bonds between diverse metal atoms, which subsequently affect the electronic environment and surface active sites in relevant reactions that are crucial for catalytic and sensor applications [53,54].

Therefore, we propose the use of thin films of IMCs as precursors for oxide-based composite nanomaterials. Our approach can allow us to control the dispersion, starting from the atomic level, resulting in a well-dispersed final multiphase material with compositional uniformity. On the other hand, further processing of the precursor can lead to the process of self-disproportionation of one component (crystallite phase) into other ones. As a consequence, interesting multiphase systems (e.g., the inclusion of metallic nanocrystals in the dielectric phase) can be obtained.

In this paper, we focus on the formation process of thin films of nanoparticles produced through the oxidation of AuSn and AuSn${}_{2}$ intermetallic compounds. We show that the composition (the atomic ratio of Au and Sn atoms) of the precursor film is the crucial parameter for the creation of Au-SnO${}_{x}$ layers of nanoparticles during the high-temperature oxidation (800 ${}^{\circ }$C). Moreover, we show that metal oxide nanoparticles that have a morphology that is desirable for catalytic applications (metal nanoparticles embedded into the oxide shell) can be produced through the oxidation of the AuSn intermetallic compound.

The difference between our proposed pathway and wet chemical procedures lays mainly in the simplicity of our proposal, especially in the context of the precursors applied and the number of reagents used. The synthesis of a bimetallic thin film by using the wet method can be implemented, for example, in three stages [6,17,33,34,35]. The first one can be realized during the synthesis of nanoparticles of one metal in a liquid phase through the chemical reduction of a precursor (e.g., for gold nanoparticles, by using a chloroauric acid solution) in the presence of stabilizing/caping and reduction agents. Then, other metal/oxide nanoparticles can be fabricated in a colloidal Ag or Au dispersion through a subsequent reduction/hydrolysis process to coat noble-metal particles. Finally, the bimetallic nanoparticles can be deposited onto a substrate. Specific preparation methods can differ substantially from this scheme, but wet chemical methods are implemented by using many chemicals that are necessary for reduction, precipitation, pH stabilization, and dispersion. In contrast, we start from two metallic precursors to obtain continuous bimetallic films that can be easily oxidized.

We believe that the proposed approach can be beneficial in more than catalysis and sensor design. The precursor is fabricated in the form of thin films deposited on a substrate (Si) through the the evaporation method. The advantage of such a strategy is that, in contrast to the post-synthetic deposition of nanoparticles that are dispersed in solution, nanostructures that are well bonded to the substrate and evenly distributed will be present from the first step of the processing. The synthesis of new composite materials (proposed in our paper in the form of thin films) responds to part of the demand for the functionalization of novel materials and surfaces [55,56].

## 2. Materials and Methods

### 2.1. Sample Preparation

Single-crystal Si(100) wafers were used as substrates for all of the samples. Gold and tin were deposited with the physical vapor deposition (PVD) process from effusion cells (Prevac EF40C1; Rogow, Poland; Al${}_{2}$O${}_{3}$ crucibles) located in a preparation chamber (base pressure: ≤$5\times 10^{-10}$ mbar). During the deposition, the substrates were kept at room temperature. The purity of the evaporated elements was 4 N for gold and 5 N for tin (both from Sigma-Aldrich; Saint Louis, MO, USA). The average deposition rate of tin was 2 Å min${}^{-1}$; for gold, the rate was 0.85 Å min${}^{-1}$. The rate and the thickness of the films were controlled with a standard quartz microbalance.

**Figure 1:**
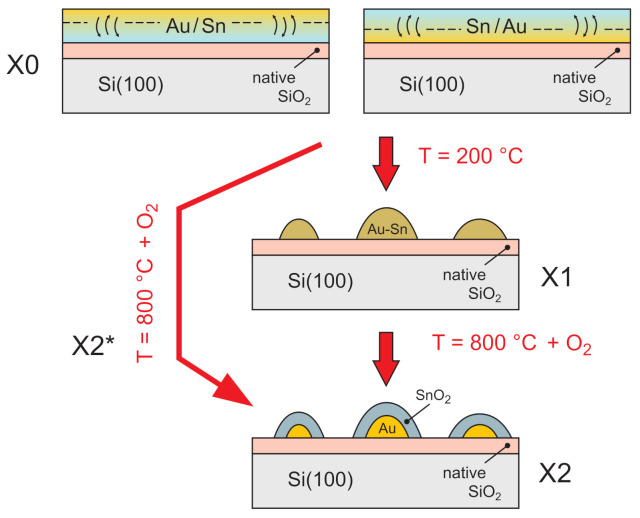
A scheme of the method of fabrication of nanostructural thin films through the oxidation of Au–Sn intermetallic compounds. X = A, B, C, or D.

Thin films were subsequently deposited—first tin, then gold (Au/Sn), or in the opposite order—with tin upon gold (Sn/Au) (see Figure 1; X0). Two sets of thicknesses were investigated: Sn (3 nm) and Au (2 nm), as well as Sn (4 nm) and Au (1 nm). The thickness of the metallic film was adjusted to obtain the desirable ratio of An to Sn atoms (Au:Sn = 1:1 for the Au (2 nm)–Sn (3 nm) and Au:Sn = 1:2 for Au (1 nm)–Sn (4 nm)). After deposition, the samples were oxidized in order to create gold particles covered by the tin oxide shell. Samples were heated in oxygen ($1\times 10^{-5}$ mbar) to 800 ${}^{\circ }$C for 8 hours (see Figure 1; X2/X2*). Most of the samples were pre-heated to 200 ${}^{\circ }$C for two hours in a vacuum in order to create Au–Sn particles before the oxidation process (see Figure 1; X1). All of the samples produced by these procedures are summarized in Table 1. In the following sections of the paper, for the as-deposited Au–Sn layers, we apply the designation X0; for those annealed at 200 ${}^{\circ }$C, we use X1; for those that were oxidized, we use X2 or X2* (see Figure 1). When referring to a specific specimen, we use markings in accordance with Table 1.

### 2.2. Sample Characterization

After deposition and/or oxidation, the samples were transferred directly (without venting) to the analysis chamber in order to perform XPS measurements. The base pressure in the analysis chamber was ≤$2\times 10^{-10}$ mbar. Monochromatic Al K${}_{\alpha }$ radiation with an energy of 1486.6 eV that was emitted at 55 degrees with respect to the normal of the samples was used as an excitation source. Photoelectron spectra were obtained by using a hemispherical VG-Scienta R3000 (Uppsala, Sweden) energy analyzer with the energy step of ΔE = 100 meV. In order to perform quantitative analysis, the experimental data from gold and tin were fitted to asymmetric line shapes by using the CasaXPS^®^ software (version 2.3.16, Casa Software Ltd., Teignmouth, UK). The data from the other components, i.e., oxygen, silicon, and carbon, were fitted by using standard Gauss–Lorentz shapes.

The ellipsometric measurements of the produced samples were performed by using the V-VASE device from J. A. Woollam Co., Inc. (Lincoln, NE, USA). The ellipsometric azimuths, Ψ and Δ, were recorded for three angles of incidence (65${}^{\circ }$, 70${}^{\circ }$, and 75${}^{\circ }$) in the spectral range from 191 to 2066 nm (0.6–6.5 eV).

Scanning electron microscopy (SEM) measurements were performed by using a Quanta 3D FEG (FEI, Hillsboro, OR, USA) (EHT = 30 kV) instrument. The samples were analyzed without the additional conductive coating. Transmission electron microscopy (TEM) and selected-area electron diffraction (SAED) were measured with an FEI (Hillsboro, OR, USA) instrument (FEI G2 F20X-Twin 200 kV, FEG) equipped with an energy-dispersive X-ray detector (EDAX, RTEM model SN9577, energy resolution: ≤136 eV). An analysis was performed in the bright-field mode and in the high-angle annular dark-field mode (for EDX). The material of the layers was collected by gently touching the copper grid surface on the analyzed samples. The grid was placed on an Si plate with a film and covered with another clean silicon plate. Then, with the grid between them, the plates were gently pressed and shifted in relation to each other to transfer the nanoparticles.

## 3. Results

The intermetallic compound layers with a total mass thickness of 5 nm were deposited from effusion cells. The thickness of the gold (2 nm for Au:Sn = 1:1 or 1 nm for Au:Sn = 1:2) and tin (3 nm for Au:Sn = 1:1 or 4 nm for Au:Sn = 1:2) thin films was established to produce IMCs with the desired composition (AuSn and AuSn${}_{2}$) [44,45]. The intermetallic compounds were formed during deposition as a result of the interphase diffusion process [44,45,57,58,59] (see Figure 1; X0). This phenomenon was observed for much thicker (than 5 nm) thin films, so we are convinced that the produced layers exhibited well-dispersed material with compositional uniformity [44,45,49].

**Figure 2:**
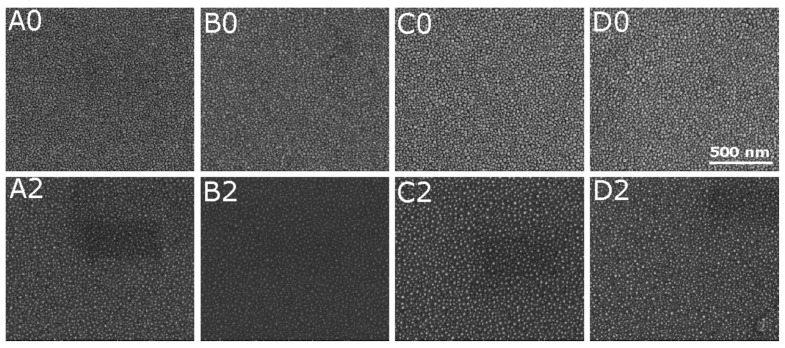
SEM images of the produced samples: as-prepared samples (X0) and samples heated at 200 ${}^{\circ }$C and then oxidized at 800 ${}^{\circ }$C (X2), where: X = A, B, C or D.

Figure 2 presents SEM images of the nanoparticles produced. The images of the un-annealed samples (A0–D0) exhibit the granular structure of the Au–Sn films with the lateral size of 15–30 nm, wherein the shape of a selected grain is not spherical.

**Figure 3:**
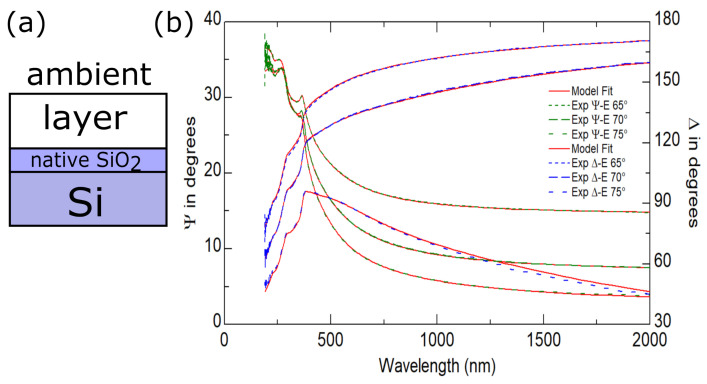
(**a**) Optical model of a sample. (**b**) Ψ and Δ azimuths (experimental and calculated data) for the B1 (Si\Sn(31)Au(19), Au:Sn = 1:1, $T_{a}$ = 200 ${}^{\circ }$C) specimen. The $\chi^{2}$ value is 4.7.

Taking into account the topography of the layers produced, for the analysis of ellipsometric data, we used a four-medium optical model of a sample (from bottom to top: Si\native SiO${}_{2}$\layer\ambient; see Figure 3a), where the optical constants of a layer were described as a Bruggeman effective medium approximation (BEMA) [60,61]:

(1) $$f_{v}\frac{n_{v}^{2}-{\tilde{n}}_{eff}^{2}}{n_{v}^{2}+2{\tilde{n}}_{eff}^{2}}+\left(1-f_{v}\right)\frac{{\tilde{n}}^{2}-{\tilde{n}}_{eff}^{2}}{{\tilde{n}}^{2}+2{\tilde{n}}_{eff}^{2}}=0.$$

In Equation (1), $f_{v}$ is the fraction of void in the layer (set to 0.5), and $n_{v}$ ($n_{v}$ = 1), ${\tilde{n}}_{m}$, and ${\tilde{n}}_{eff}$ are the refractive indexes of the ambient, material, and effective medium (effective layer), respectively. Generally, the complex refractive index is a complex quantity:

(2) $$\tilde{n}=n-ik,$$

where *n* is the real part of $\tilde{n}$, *k* is the extinction coefficient, and *i* is an imaginary unit. Based on the determined spectra of the extinction coefficient, the absorption coefficient was calculated by using the following relation: $\alpha =\frac{2\pi k}{\lambda }$, where λ is the extinction coefficient.

The optical constants of the produced films were parametrized by using a sum of three or four Gaussian oscillators [60,61]:

(3) $${\tilde{n}}^{2}=\varepsilon_{\infty }+\sum_{j}Gauss\left(A_{j},E_{j},Br_{j}\right),$$

where $\varepsilon_{\infty }$ is a high-frequency dielectric constant, while $A_{n}$, $E_{n}$, and $Br_{n}$ are the amplitude, energy, and broadening of the *j*-th oscillator, respectively. The optical constants of Si and SiO${}_{2}$ were taken from the database of optical constants [60].

The model parameters (the thickness of a layer and quantities in Equation (3)) were adjusted to minimize the reduced mean squared error ($\chi^{2}$), which was defined as [60,61]:

(4) $$\chi^{2}=\frac{1}{N-P}\sum_{j}\left(\frac{{\left(\Psi_{j}^{mod}-\Psi_{j}^{exp}\right)}^{2}}{\sigma_{\Psi \;j}^{2}}+\frac{{\left(\Delta_{j}^{mod}-\Delta_{j}^{exp}\right)}^{2}}{\sigma_{\Delta \;j}^{2}}\right),$$

where *N* and *P* are the total number of data points and the number of fitted model parameters, respectively. In Equation (4), the quantities $\Psi_{j}^{exp}$, $\Delta_{j}^{exp}$, $\Psi_{j}^{mod}$, and $\Delta_{j}^{mod}$ are the experimental (with superscript “exp”) and calculated (with superscript “mod”) ellipsometric azimuths. The quantities $\sigma_{\Psi \;j}$ and $\sigma_{\Delta \;j}$ are the standard deviations determined for the Ψ and Δ azimuths. An example of the fit is presented in Figure 3b. The determined values of $\chi^{2}$ were lower than 4.7 for all of the samples examined in this study.

**Figure 4:**
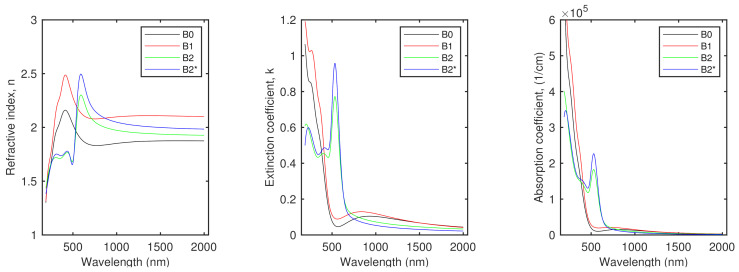
Refractive index (*n*), extinction coefficient (*k*), and absorption coefficient (α) determined for series B. Spectra for the other samples are presented in the Supplementary Information.

Table 2 shows the effective thickness of the fabricated intermetallic thin films directly after deposition (samples X0) and after annealing in a vacuum at 200 ${}^{\circ }$C (samples X1). The thickness of a layer was 2.5–3.5 times larger than that obtained based on the quartz crystal microbalance. The total thickness of 5 nm was established by assuming that the layer was smooth and that its density was equal to the density of a bulk material. These discrepancies were associated with the granular structure of the Au–Sn films produced (see Figure 2). The optical constants of the obtained intermetallic compound layers, which are presented in Figure 4 and Figure S1 (see Supplementary Information), confirm their granular structure. The high values of *k* (and α) in the UV spectral range for the samples that were deposited (X0) and annealed in a vacuum (X1) were related to the inter-band transitions of the AuSn (the A and B series of samples) and AuSn${}_{2}$ (the C and D series of samples) IMCs [44,45]. In the IR spectral range, the annealing at 200 ${}^{\circ }$C led to an increase in the refractive index value. This change can be assigned to the formation of a more dense structure of the material. The absorption features in the NIR spectral range with a wide maximum at 750–850 nm (for the C0 and C1 specimens, the maximum was not evident) were associated with the granular structure of a layer, as was observed earlier [44,45,49,62]. However, the most prominent feature of these spectra that confirmed the granular structure of the Au–Sn films produced was the lack of the Drude term [61], i.e., the lack of absorption in the IR spectral range related to the interaction of the electromagnetic radiation with the free carriers of a conductor—both *k* and α for the long wavelengths were close to zero.

The oxidation of the thin film of the AuSn intermetallic compound at 800 ${}^{\circ }$C led to the formation of uniformly distributed structures with a size of ∼20–30 nm (see Figure 2). The optical responses of these structures differs significantly from those of the un-annealed samples. First of all, a strong absorption band was visible at ∼530 nm for the A and B series of samples and at about ∼520 nm for the C and D systems (see the *k* and α spectra in Figure 4 and Figure S1—see Supplementary Information). This strong absorption is typical for metallic nanoparticles [17] (also in the dielectric matrix [6,17]) and is described as the plasmonic effect, i.e.,the interaction of the incident electromagnetic wave with free electrons in the the metal. Moreover, the decrease in the extinction coefficient (and absorption coefficient) to zero with the increase in the wavelength of the incident light and the normal dispersion of the refractive index in the IR spectral range point to the semiconducting behavior of the structures produced.

**Figure 5:**
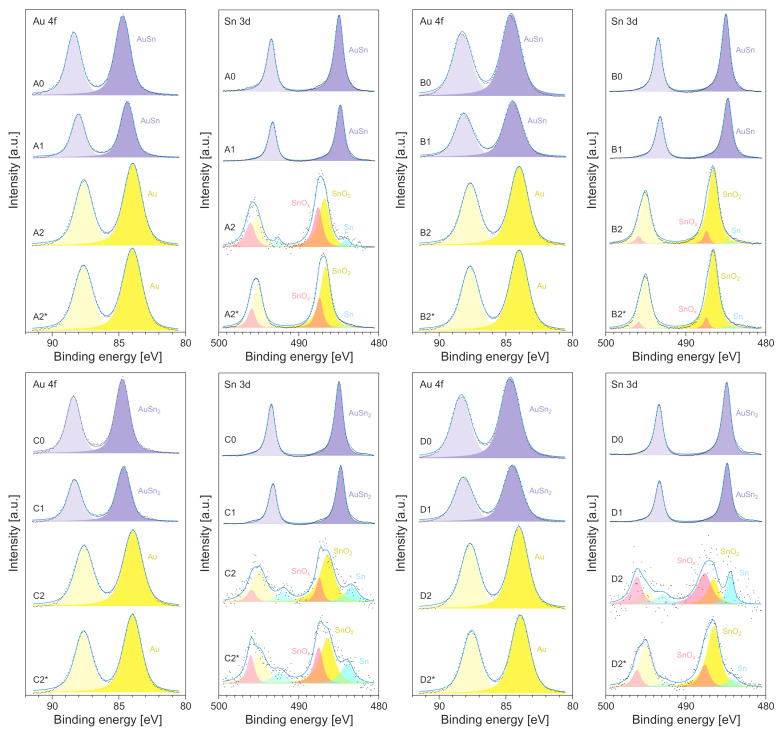
X-ray photoelectron spectroscopy (XPS) spectra (Au 4f and Sn 3d peaks) of the samples: X0—as prepared, X1—heated at 200 ${}^{\circ }$C, X2—heated at 200 ${}^{\circ }$C and then oxidized at 800 ${}^{\circ }$C, and X2*—oxidized at 800 ${}^{\circ }$C (without the heating at 200 ${}^{\circ }$C), where X = A, B, C, or D.

To examine the chemical state of the synthesized nanostructures, XPS measurements were performed. The results for all of the series of samples are presented in Figure 5. Figure 5 shows a typical XPS signal of Au 4f and Sn 3d. For all of the as-deposited samples (X0), the Au doublet (Au 4f${}_{5/2}$ and Au 4f${}_{7/2}$) registered for the AuSn and AuSn${}_{2}$ IMCs was shifted by about 0.7 eV towards higher energies compared to the Au${}^{0}$ signal, which should have been centered at 84.0 eV (for Au 4f${}_{7/2}$) [63]. The formation (during the deposition process) of the bimetallic compound was also confirmed in the Sn 3d doublet (slightly shifted by 1.0 eV towards higher energies). For the metallic tin (Sn${}^{0}$), the Sn 3d${}_{5/2}$ component should have been centered at 484.0 eV [64]. For the X0 samples, this peak was observed at 485.0 eV. The annealing of the X0 specimen in a vacuum basically did not change the shapes of the XPS spectra (see Figure 5). This means that the X1 samples did not change their chemical state.

The XPS spectra recorded for the oxidized systems (the X2/X2* samples) differed significantly from those obtained for the as-deposited (X0) and annealed (X1) specimens. Firstly, the position of the single Au 4f doublet corresponded to metallic gold (Au${}^{0}$), i.e., the Au 4f${}_{7/2}$ peak was at 84.0 eV for all of the oxidized samples (see Figure 5). Secondly, the Sn 3d peak could be deconvoluted into three components. Two components of Sn 3d${}_{5/2}$ at the positions 487.4–487.6 eV and at 486.5–486.8 eV could be assigned to oxidized states of tin (Sn${}^{4+/2+}$) [65], wherein the signal at a higher energy was associated with the SnO${}_{2}$ oxide (Sn${}^{4+}$) [65]. The third component of the Sn 3d${}_{5/2}$ peak centered at 484 eV could be assigned to metallic tin (Sn${}^{0}$) [64]; however, its intensity was negligible. It should be noted that the Sn 3d signal obtained for the oxidized AuSn${}_{2}$ compound (the C2/C2* and D2/D2* samples) exhibitde a quite low intensity (compared to the results obtained for the oxidized AuSn intermetallic compound); see Figure 5. After oxidation, the ratio of Au to Sn atoms was at the level of 10:1. This result differed significantly from the expected ratio of Au:Sn = 1:2. This effect indicates that the tin atoms sublimated from the surface of the sample. For the AuSn intermetallic compound, the ratio of Au to Sn atoms was close to the assumed value (Au:Sn = 1:1). The difference in the behaviors of the tin atoms in AuSn and AuSn${}_{2}$ during the oxidation at 800 ${}^{\circ }$C can be explained as a difference in their melting and vaporization temperatures [66]. Lower values were exhibited the Sn-rich compound. This result was confirmed in the spectroscopic ellipsometry investigations. The thickness of the oxidized systems from the C and D series of samples (C2/C2* and D2/D2*) was estimated to be about 5 nm, which was 3–4 times thinner (see Table 2) than that determined for the non-oxidized samples and those oxidized based on the AuSn intermetallic compound.

**Figure 6:**
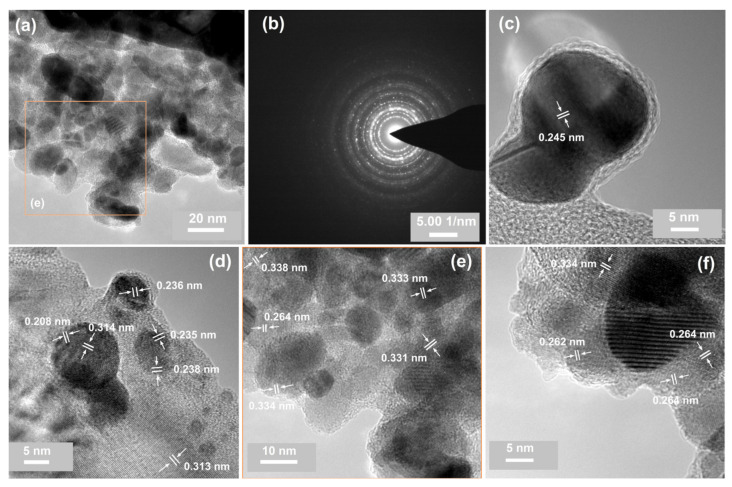
TEM characterization of the samples from the A and B series: (**a**) a TEM image at a lower magnification of a Sn/Au-rich material of sample B2*; (**b**) a selected-area electron diffraction (SAED) pattern of sample B2*; (**c**) a high-resolution TEM image presenting individual Au nanoparticles registered for sample A2*; (**d**) a high-resolution TEM image illustrating a Au/Si-rich area of sample A2; (**e**) a high-resolution TEM image of a selected area of (**a**); (**f**) a high-resolution TEM image of a bimetallic fragment of sample B2*; Moiré fringes are visible.

The analysis of the TEM results revealed that the decomposition sequence, the thermal treatment mode, and the initial phase composition of the layer had an impact on the structural and compositional characteristics of the samples. It should be noted that some morphological features derived from the TEM results can be attributed to the fragmentation of the materials in the layers during the preparation of the specimens for TEM characterization. The EDX spectra (see Figure S2 and Table S1, Supplementary Information) exhibited only the traces of tin for the samples from the C and D series. In the case of the A and B samples, Au-, Sn-, and Au/Sn-rich specimens were found. Moreover, the EDX analysis of these samples pointed to the higher silicon content in the film fragments that contained only gold atoms. The existence of Si was most probably associated with the process of collection of nanoparticles (by touching the grid surface on the analyzed samples). This suggests the Au-rich composition of the bottom part of the heated bimetallic films. The EDX results also indicated a partial separation of the Au- and Sn-containing phases. The SnO${}_{2}$ phase was often visible as a form of separate crystallites (Figure 6a,e), while the Au crystallites were widely surrounded by an amorphous form, most probably of SnO${}_{x}$ (Figure 6c,d). It should be noted that SnO${}_{x}$-coated (2–3 nm) Au crystallites with a size of 5–10 nm (Au@SnO${}_{x}$) are desirable in catalytic applications [17,33]. This result agreed well with the XPS results, where for the Sn 3d peak, two oxide components were found (SnO${}_{2}$ and SnO${}_{x}$; see Figure 5). Areas that simultaneously included the two metals were a significant part of the studied system for samples A2* (Figure 6f) and B2* (Figure 6a,b,e), which were heated directly at 800 ${}^{\circ }$C.

The bimetallic material that was detached from the substrates of samples A2* and B2* was composed of nano-sized crystals that were randomly oriented and embedded in an amorphous environment (Figure 6e,f). The high-magnification TEM images registered for the A and B series indicated lattice distances of 0.334 and 0.264 nm, which corresponded well to the d-spacing from the tetragonal SnO${}_{2}$ (110) and (101) planes [67]. Moiré fringes with a periodicity of around 0.8 nm were also visible at the highest magnification as a result of the overlapping crystalline sections of the sample (Figure 6f). Simultaneously, the TEM image analysis did not allow the localization of the gold-containing phase detected in a bimetallic area by EDX. This suggests that the gold particles were surrounded by a tin-containing phase (Figure 6e,f). The appropriate selected-area electron diffraction (SAED) results (Figure 6f) exhibited sharp single crystalline spots and polycrystalline rings from tin(IV) oxide [67] and metallic gold [68] (Figure 6b; Table S2, see Supplementary Information). Additionally, the overall TEM results pointed to the greater separation of both metal-containing phases for samples A and B, which were thermally treated at 200 ${}^{\circ }$C and then oxidized (Figure 6c,d).

**Figure 7:**
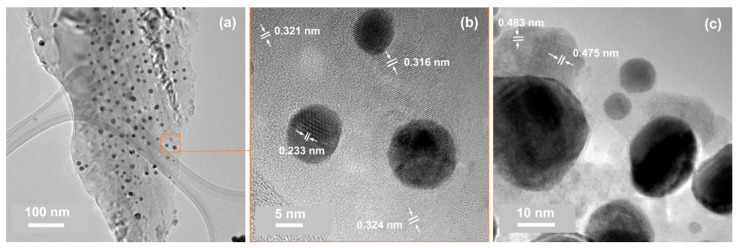
TEM characterization of samples from the C and D series: (**a**) a TEM image of sample D2* at a lower magnification; (**b**) a high-resolution TEM image of a selected area of (**a**); (**c**) a high-resolution TEM image illustrating Au grains and SnO nanocrystals (upper-left corner).

The EDX spectra (Figure S2, Table S1, Supplementary Information) exhibited only traces of tin for the samples from the C and D series. This result confirmed the outcome of the XPS measurements performed for the C and D series of samples (see Figure 5), where the Sn 3d signal exhibited a significantly lower intensity than that recorded for the A and B specimens. This effect can be explained as a consequence of the sublimation of (mostly) tin atoms from the surface of the sample (see Table 2; the difference in thickness of X0/X1 and X2/X2* for the C and D series of samples). The TEM results (Tables S1 and S3) confirmed that the analyzed material consisted of gold and silicon [69], and only isolated spots or rings from SnO${}_{2}$ or SnO were observed. For example, the high-resolution TEM image of one area (Figure 7c) showed an interplanar spacing consistent with the (001) crystal face of SnO nanocrystals [70]. The quasi-spherical gold nanoparticles detected through TEM ranged from 5 to 20 nm (Figure 7a–c). The data from the literature indicate that the high-temperature annealing of gold–silicon systems can lead to the diffusion of Au nanoparticles or even to the formation of gold silicides in the nanoparticles [71,72,73,74]. Thus, the estimated d-spacing of ca. 0.32 nm for Si (Figure 7b and Figure 6d) could be a result of this process. However, the XPS measurements (see Figure 5) did not indicate this component. It was demonstrated that the native SiO${}_{2}$ significantly reduced the process of gold silicate formation [71].

## 4. Conclusions

Spectroscopic ellipsometry (SE), X-ray photoelectron spectroscopy (XPS), and transmission electron microscopy (TEM) combined with energy-dispersive X-ray spectroscopy (EDX) were used to investigate the process of nanoparticle formation through the oxidation of AuSn and AuSn${}_{2}$ thin films and to characterize the obtained products. The precursors were produced through the sequential deposition of gold and tin and as an effect of interdiffusion of Au and Sn atoms. We found that the sequence of deposition exerted a less significant impact on the final result than the atomic ratio between Au and Sn atoms, which was found to be the crucial parameter for the process of nanoparticle production. The sizes of all of the fabricated nanoparticles were below 30 nm. Optical measurements performed for the oxidized samples exhibited a strong absorption peak at about 520–530 nm, which was typical for metallic nanostructures (the plasmonic effect). The oxidation of the AuSn compound (the atomic ratio of Au and Sn atoms of 1:1) led to the formation of a mixture of Au@SnO${}_{x}$ (amorphous SnO${}_{x}$) and SnO${}_{2}$ (crystallite phase) nanoparticles. The oxidation of the AuSn${}_{2}$ intermetallic compound at 800 ${}^{\circ }$C led to the sublimation of almost all of the tin atoms. For these systems, (primarily) Au nanocrystallites were found.

## Figures and Tables

**Table 1:** The samples produced: id., layered structure of a sample, the atomic ratio (Au:Sn), the temperature of annealing ($T_{a}$), and the temperature of oxidation ($T_{o}$).

No.	Id.	Sequence of Deposition	Au:Sn	$T_{a}$ (°C)	$T_{o}$ (°C)
1.	A0	Si\Au(19)\Sn(31)	1:1	-	-
2.	A1	Si\Au(19)\Sn(31)	1:1	200	-
3.	A2	Si\Au(19)\Sn(31)	1:1	200	800
4.	A2*	Si\Au(19)\Sn(31)	1:1	-	800
5.	B0	Si\Sn(31)\Au(19)	1:1	-	-
6.	B1	Si\Sn(31)\Au(19)	1:1	200	-
7.	B2	Si\Sn(31)\Au(19)	1:1	200	800
8.	B2*	Si\Sn(31)\Au(19)	1:1	-	800
9.	C0	Si\Au(12)\Sn(38)	1:2	-	-
10.	C1	Si\Au(12)\Sn(38)	1:2	200	-
11.	C2	Si\Au(12)\Sn(38)	1:2	200	800
12.	C2*	Si\Au(12)\Sn(38)	1:2	-	800
13.	D0	Si\Sn(38)\Au(12)	1:2		-
14.	D1	Si\Sn(38)\Au(12)	1:2	200	-
15.	D2	Si\Sn(38)\Au(12)	1:2	200	800
16.	D2*	Si\Sn(38)\Au(12)	1:2	-	800

**Table 2:** The effective thickness ($d_{eff}$) and the energy of the absorption peaks ($E_{max}$).

No.	Id.	$d_{\mathrm{eff}}$ (nm)	$E_{\max}$ (nm)	$E_{\max}$ (eV)
1.	A0	18.1 ± 0.9	-	-
2.	A1	17.4 ± 1.6	-	-
3.	A2	8.3 ± 0.1	530 ± 4	2.34 ± 0.02
4.	A2*	18.5 ± 0.1	530 ± 5	2.34 ± 0.02
5.	B0	14.3 ± 0.8	-	-
6.	B1	14.2 ± 0.9	-	-
7.	B2	10.2 ± 0.2	530 ± 5	2.34 ± 0.02
8.	B2*	14.4 ± 0.1	530 ± 5	2.34 ± 0.02
9.	C0	15.5 ± 0.6	-	-
10.	C1	16.5 ± 0.8	-	-
11.	C2	4.8 ± 0.1	521 ± 4	2.38 ± 0.02
12.	C2*	4.0 ± 0.1	521 ± 4	2.38 ± 0.02
13.	D0	13.5 ± 0.5	-	-
14.	D1	14.1 ± 0.6	-	-
15.	D2	4.5 ± 0.1	521 ± 4	2.38 ± 0.02
16.	D2*	5.3 ± 0.1	521 ± 4	2.38 ± 0.02

## Data Availability

Data are available from the corresponding author.

## Supplementary Materials

The following are available online at https://www.mdpi.com/article/10.3390/ma14144034/s1, Figure S1: Refractive index (*n*), extinction coefficient (*k*) and absorption coefficient (*α*) determined for all the investigated samples, Figure S2: Energy dispersive X-ray spectroscopy analysis (EDX spectrum and corresponding STEM-HAADF image): (a) sample A2; (b) sample A2* (low magnification image including area presented in Figure 6a,e; (b) sample B2*; (b) sample C2 (low magnification image including area presented in Figure 7c), Table S1: Results of the EDX analysis of the samples, Table S2: Indexed Lattice Planes from the SAED pattern presented in Figure 6f, Table S3: Indexed Lattice Planes from the SAED pattern registered for sample C2.

## References

[1] Zhang Y., Li l., Ao S., Wang J., Li G. (2018). Interfacial Doping of Heteroatom in Porous SnO_2_ for Highly Sensitive Surface Properties. ACS Omega.

[2] Wang X., Tian J.S., Zheng Y.H., Xu X.L., Liu M.W., Fang X.X. (2014). Tuning Al_2_O_3_ Surface with SnO_2_ to Prepare Improved Supports for Pd for CO Oxidation. ChemCatChem.

[3] Li Z., Li H., Wu Z., Wang M., Luo J., Torun H., Hu P., Yang C., Grundmann M., Liu X. (2019). Advances in designs and mechanisms of semiconducting metal oxide nanostructures for high-precision gas sensors operated at room temperature. Mater. Horiz..

[4] Cheng J.P., Wang B.B., Zhao M.G., Liu F., Zhang X.B. (2014). Nickel-doped tin oxide hollow nanofibers prepared by electrospinning for acetone sensing. Sens. Actuators B.

[5] Ding L.X., Wang A.L., Ou Y.N., Li Q., Guo R., Zhao W.X., Tong y.X., Li G.R. (2013). Hierarchical Pd-Sn alloy nanosheet dendrites: An economical and highly active catalyst for ethanol electrooxidation. Sci. Rep..

[6] Liu W.-L., Lin F.C., Yang Y.-C., Huang C.-H., Gwo S., Huang M.H., Huang J.-S. (2013). The influence of shell thickness of Au@TiO_2_ core–shell nanoparticles on the plasmonic enhancement effect in dye-sensitized solar cells. Nanoscale.

[7] Hosseini Z.S., Zad A.I., Mortezaali A. (2015). Room temperature H_2_S gas sensor based on rather aligned ZnO nanorods with flower-like structures. Sens. Actuators B.

[8] Song W., Wu H., Wang J., Lin Y., Song J., Xie Y., Li L., Shi K. (2015). A CH_3_NH_3_PbI_3_ film for a room-temperature NO_2_ gas sensor with quick response and high selectivity. Aust. J. Chem..

[9] Wang Y., Duan G., Zhu Y., Zhang H., Xu Z., Dai Z., Cai W. (2016). Room temperature H_2_S gas sensing properties of In_2_O_3_ micro/nanostructured porous thin film and hydrolyzation-induced enhanced sensing mechanism. Sens. Actuators B.

[10] Bhati V.S., Ranwa S., Rajamani S., Kumari K., Raliya R., Biswas P., Kumar M. (2018). Improved sensitivity with low limit of detection of a hydrogen gas sensor based on rGO-loaded Ni-doped ZnO nanostructures. ACS Appl. Mater. Interfaces.

[11] Kou X., Xie N., Chen F., Wang T., Guo L., Wang C., Wang Q., Ma J., Sun Y., Zhang H. (2018). Superior acetone gas sensor based on electrospun SnO_2_ nanofibers by Rh doping. Sens. Actuators B.

[12] Kim H., Pak Y., Jeong Y., Kim W., Kim J., Jung G.Y. (2018). Amorphous Pd–assisted H_2_ detection of ZnO nanorod gas sensor with enhanced sensitivity and stability. Sens. Actuators B.

[13] Sankar Ganesh R., Navaneethan M., Patil V.L., Ponnusamy S., Muthamizhchelvan C., Kawasaki S., Patil P.S., Hayakawa Y. (2018). Sensitivity enhancement of ammonia gas sensor based on Ag/ZnO flower and nanoellipsoids at low temperature. Sens. Actuators B.

[14] Park S., Kim S., Kheel H., Hyun S.K., Jin C., Lee C. (2016). Enhanced H_2_S gas sensing performance of networked CuO–ZnO composite nanoparticle sensor. Mater. Res. Bull..

[15] Gao H., Zhao L., Wang L., Sun P., Lu H., Liu F., Chuai X., Lu G. (2018). Ultrasensitive and low detection limit of toluene gas sensor based on SnO_2_-decorated NiO nanostructure. Sens. Actuators B.

[16] Chen H., Hu J., Li G.-D., Gao Q., Wei C., Zou X. (2017). Porous Ga–In Bimetallic Oxide Nanofibers with Controllable Structures for Ultrasensitive and Selective Detection of Formaldehyde. ACS Appl. Mater. Interfaces.

[17] Cai Z., Wu Y., Wu Z., Yin L., Weng Z., Zhong Y., Xu W., Sun X., Wang H. (2018). Unlocking Bifunctional Electrocatalytic Activity for CO_2_ Reduction Reaction by Win-Win Metal–Oxide Cooperation. ACS Energy Lett..

[18] Fang M., Dong G., Wei R., Ho J.C. (2017). Hierarchical Nanostructures: Design for Sustainable Water Splitting. Adv. Energy Mater..

[19] Grimaud A., Diaz-Morales O., Han B., Hong W.T., Lee Y.-L., Giordano L., Stoerzinger K.A., Koper M.T.M., Shao-Horn Y. (2017). Activating lattice oxygen redox reactions in metal oxides to catalyse oxygen evolution. Nat. Chem..

[20] Wang X., He Y., Liu C., Liu Y., Qiao Z.-A., Huo Q. (2016). A controllable asymmetrical/symmetrical coating strategy for architectural mesoporous organosilica nanostructures. Nanoscale.

[21] Jo Y.-M., Kim T.-H., Lee C.-S., Lim K., Na C., Abdel-Hady F., Wazzan A.A., Lee J.-H. (2018). Metal–Organic Framework–Derived Hollow Hierarchical Co_3_O_4_ Nanocages with Tunable Size and Morphology: Ultrasensitive and Highly Selective Detection of Methylbenzenes. ACS Appl. Mater. Interfaces.

[22] Choi J., Kim W.-S., Hong S.-H. (2018). Highly stable SnO_2_-Fe+2O_3_-C hollow spheres for reversible lithium storage with extremely long cycle life. Nanoscale.

[23] Rai P., Yoon J.-W., Jeong H.-M., Hwang S.-J., Kwak C.-H., Lee J.-H. (2014). Design of highly sensitive and selective Au@NiO yolk-shell nanoreactors for gas sensor applications. Nanoscale.

[24] Ding H., Zhang Y., Xu S., Li G. (2016). A wrinkle to sub–100 nm yolk/shell Fe_3_O_4_@SiO_2_ nanoparticles. Nano Res..

[25] Zhang Q., Wang J., Dong J., Ding F., Li X., Zhang B., Yang S., Zhang K. (2015). Facile general strategy toward hierarchical mesoporous transition metal Oxides Arrays on three-dimensional macroporous foam with superior lithium storage Properties. Nano Energy.

[26] Zheng N., Stucky G.D. (2006). A General Synthetic Strategy for Oxide–Supported Metal Nanoparticle Catalysts. J. Am. Chem. Soc..

[27] Guo T., Yao M.-S., Lin Y.-H., Nan C.-W. (2015). A comprehensive review on synthesis methods for transition-metal oxide nanostructures. CrystEngComm.

[28] Zhang Q., Zhang K., Xu D., Yang G., Huang H., Nie F., Liu C., Yang S. (2014). CuO nanostructures: Synthesis, characterization, growth mechanisms, fundamental properties, and applications. Prog. Mater. Sci..

[29] Yuan C., Wu H.B., Xie Y., Lou X.W. (2014). Mixed Transition–Metal Oxides: Design, Synthesis, and Energy-Related Applications. Angew. Chem. Int. Ed..

[30] Carretero-Genevriera A., Mestres N. (2015). Growth of 1-D Oxide Nanostructures. Encyclopedia of Nanotechnology.

[31] Liang Y., Chen Z., Yao W., Wang P., Yu S., Wang X. (2017). Decorating of Ag and CuO on Cu Nanoparticles for Enhanced High Catalytic Activity to the Degradation of Organic Pollutants. Langmuir.

[32] Bertuna A., Faglia G., Ferroni M., Kaur N., Arachchige H.M.M.M., Sberveglieri G., Comini E. (2017). Metal Oxide Nanowire Preparation and Their Integration into Chemical Sensing Devices at the SENSOR Lab in Brescia. Sensors.

[33] Yu K., Wu Z., Zhao Q., Li B., Xie Y. (2008). High-Temperature-Stable Au@SnO_2_ Core/Shell Supported Catalyst for CO Oxidatio. J. Phys. Chem. C.

[34] Louis C. (2016). Chemical Preparation of Supported Bimetallic Catalysts. Gold-Based Bimetallic, a Case Study. Catalysts.

[35] Pan X., Zheng J., Zhang L., Yi Z. (2019). Core-Shell Au@SnO_2_ Nanostructures Supported on Na_2_Ti_4_O_9_ Nanobelts as a Highly Active and Deactivation-Resistant Catalyst toward Selective Nitroaromatics Reduction. Inorg. Chem..

[36] Ismail A.M., Samu G.F., Balog Á., Csapoó E., Janaáky C. (2019). Composition-Dependent Electrocatalytic Behavior of Au-Sn Bimetallic Nanoparticles in Carbon Dioxide Reduction. ACS Energy Lett..

[37] Haruta M., Kobayashi T., Sano H., Yamada N. (1987). Novel Gold Catalysts for the Oxidation of Carbon Monoxide at a Temperature far Below 0 °C. Chem. Lett..

[38] Corma A., Serna P. (2006). Chemoselective Hydrogenation of Nitro Compounds with Supported Gold Catalysts. Science.

[39] Zhong Z., Lin J., Teh S.-P., Teo J., Dautzenberg F.M. (2007). A Rapid and Efficient Method to Deposit Gold Particles on Catalyst Supports and Its Application for CO Oxidation at Low Temperatures. Adv. Funct. Mater..

[40] Zhong C.J., Maye M.M. (2001). Core–Shell Assembled Nanoparticles as Catalysts. Adv. Mater..

[41] Cui Z.M., Li L.J., Manthiram A., Goodenough J.B. (2015). A Surfactant-Free Strategy for Synthesizing and Processing Intermetallic Platinum-Based Nanoparticle Catalysts. J. Am. Chem. Soc..

[42] Cui Z.M., Chen H., Zhao M.T., Marshall D., Yu Y.C., Abruna H., DiSalvo F.J. (2014). Synthesis of structurally ordered Pt_3_Ti and Pt_3_V nanoparticles as methanol oxidation catalysts. J. Am. Chem. Soc..

[43] Kang Y.J., Pyo J.B., Ye X.C., Gordon T.R., Murray C.B. (2012). Synthesis, Shape Control, and Methanol Electro-oxidation Properties of Pt–Zn Alloy and Pt_3_Zn Intermetallic Nanocrystals. Acs Nano.

[44] Rerek T., Skowronski L., Szczesny R., Naparty M.K., Derkowska-Zielinska B. (2020). The effect of the deposition rate on morphology, opto-electronic properties and formation intermetallic compounds of Au–Sn alloys. J. Alloys Compd..

[45] Rerek T., Skowronski L., Kobierski M., Naparty M.K., Derkowska-Zielinska B. (2018). Microstructure and opto-electronic properties of Sn-rich Au-Sn diffusive solders. Appl. Surf. Sci..

[46] Wronkowska A.A., Wronkowski A., Bukaluk A., Trzcinski M., Okulewicz K., Skowronski L. (2008). Structural analysis of In/Ag, In/Cu and In/Pd thin films on tungsten by ellipsometric, XRD and AES methods. Appl. Surf. Sci..

[47] Wronkowska A.A., Wronkowski A., Skowronski L. (2009). Non-destructive characterization of In/Ag and In/Cu diffusive layers. J. Alloys Compd..

[48] Wronkowska A.A., Wronkowski A., Kukliński K., Senski M., Skowronski L. (2010). Spectroscopic ellipsometry study of the dielectric response of Au-In and Ag-Sn thin-film couples. Appl. Surf. Sci..

[49] Wronkowska A.A., Czerniak G., Wronkowski A., Skowronski L. (2013). Optical and microstructural characterisation of Au–Sn and Cu–Sn diffusive layers. Appl. Surf. Sci..

[50] Kasprzak W., Amirkhiz B.S., Niewczas M. (2014). Structure and properties of cast Al–Si based alloy with Zr–V–Ti additions and its evaluation of high temperature performance. J. Alloys Compd..

[51] Chookajorn T., Murdoch H.A., Schuh C.A. (2012). Design of Stable Nanocrystalline Alloys. Science.

[52] Mosby J.M., Prieto A.L. (2008). Direct electrodeposition of Cu_2_Sb for lithium-ion battery anodes. J. Am. Chem. Soc..

[53] Yang Y., Wei M. (2020). Intermetallic compound catalysts: Synthetic scheme, structure characterization and catalytic application. J. Mater. Chem. A.

[54] Zhang Y., Li L., Li Q., Fan J., Zheng J., Li G. (2016). Smart Solution Chemistry to Sn-Containing Intermetallic Compounds through a Self-Disproportionation Process. Chem. A Eur. J..

[55] Guizard C., Princivalle A. (2009). Preparation and characterization of catalyst thin films. Catal. Today.

[56] Mehla S., Das J., Jampaiah D., Periasamy S., Nafady A., Bhargava S.K. (2019). Recent advances in preparation methods for catalytic thin films and coatings. Catal. Sci. Technol..

[57] Sutter P., Tenney S.A., Ivars-Barcelo F., Wu L., Zhu Y., Sutter E. (2016). Alloy oxidation as a route to chemically active nanocomposites of gold atoms in a reducible oxide matrix. Nanoscale Horiz..

[58] Wang J.-G., Tian M.-L., Mallouk T.E., Chan M.H.W. (2004). Microstructure and interdiffusion of template-synthesized Au/Sn/Au junction nanowires. Nano Lett..

[59] Yamada T., Miura K., Kajihara M., Kurokawa N., Sakamoto K. (2005). Kinetics of reactive diffusion between Au and Sn during annealing at solid-state temperatures. Mater. Sci. Eng. A.

[60] Woollam J.A. (2010). Guide to Using WVASE32^®^.

[61] Fujiwara H. (2009). Spectroscopic Ellipsometry. Principles and Applications.

[62] Rerek T., Skowronski L., Szczesny R., Naparty M.K., Derkowska-Zielinska B. (2019). The effect of the deposition rate on microstructural and opto-electronic properties of *β*-Sn layers. Thin Solid Films.

[63] Sadhukhan P., Barman S., Roy T., Singh V.K., Sarkar S., Chakrabarti A., Barman S.R. (2019). Electronic structure of Au-Sn compounds grown on Au(111). Phys. Rev. B.

[64] Tang W., He A., Liu Q., Ivey D.G. (2008). Room temperature interfacial reactions in electrodeposited Au/Sn couples. Acta Mater..

[65] Taylor J.A., Merchant S.M., Perry D.L. (1995). Study of the oxidation of gold-tin performs using x-ray photoelectron spectroscopy. J. Appl. Phys..

[66] Ciulik J., Notis M.R. (1993). The Au-Sn phase diagram. J. Alloys Compd..

[67] Bolzan A.A., Fong C., Kennedy B.J., Howard C.J. (1997). Structural studies of rutile-type metal dioxides. Acta Crystallogr. Sect. B Struct. Sci..

[68] Suh I.-K., Ohta H., Waseda Y. (1988). High-temperature thermal expansion of six metallic elements measured by dilatation method and X-ray diffraction. J. Mater. Sci..

[69] Hanfland M., Schwarz U., Syassen K., Takemura K. (1999). Crystal structure of the high-pressure phase silicon VI. Phys. Rev. Lett..

[70] Izumi F. (1981). Pattern-fitting structure refinement of tin(II) oxide. J. Solid State Chem..

[71] Bhatta U.M., Dash J.K., Rath A., Satyam P.V. (2009). Structural phase transitions in Au thin films on Si (110): An in situ temperature dependent transmission electron microscopy study. Appl. Surf. Sci..

[72] Li Y., Shi W., Gupta A., Chopra N. (2015). Morphological evolution of gold nanoparticles on silicon nanowires and their plasmonics. RSC Adv..

[73] Bhatta U.M., Dash J.K., Rath A., Satyam P.V. (2009). Formation of aligned nanosilicide structures in a MBE-grown Au/Si(110) system: A real-time temperature-dependent TEM study. J. Phys. Condens. Matter.

[74] Rath A., Juluri R.R., Satyam P.V. (2014). Real time nanoscale structural evaluation of gold structures on Si (100) surface using in-situ transmission electron microscopy. J. Appl. Phys..

