# Supplementary material for: Microstructure and Optical Properties of Nanostructural Thin Films Fabricated through Oxidation of Au–Sn Intermetallic Compounds
Source: Materials (Basel). 2021 Jul 19;14(14):4034. doi: 10.3390/ma14144034 (PMC8307610; doi:10.3390/ma14144034)
Supplement: Supplementary file 1 [file materials-14-04034-s001.zip › materials-1285470-supplementary.pdf]

Supplementary Information

Microstructure and optical properties of nanostructural thin films fabricated by oxidation of Au-Sn intermetallic compounds

L. Skowronski[a,@], M. Trzcinski[a], A. Olszewska[a], R. Szczesny[b]

[a] Institute of Mathematics and Physics, UTP University of Science and Technology, Kaliskiego 7, 85-796 Bydgoszcz, Poland
[b] Faculty of Chemistry, Nicolaus Copernicus University in Torun, Gagarina 7, 87-100 Torun, Poland

[@] *lukasz.skowronski@utp.edu.pl*

This Supplementary Information includes:

Figure S1: Refractive index (n), extinction coefficient (k) and absorption coefficient (α) determined for all the investigated samples.

Figure S2: Energy dispersive X-ray spectroscopy analysis (EDX spectrum and corresponding STEM-HAADF image): (a) sample A2; (b) sample A2* (low magnification image including area presented in Fig. 6 (a), (e)); (b) sample B2*; (b) sample C2 (low magnification image including area presented in Fig.7 (c)).

Table S1: Results of the EDX analysis of the samples.

Table S2: Indexed Lattice Planes from the SAED pattern presented in Figure 6f.

Table S3: Indexed Lattice Planes from the SAED pattern registered for sample C2.

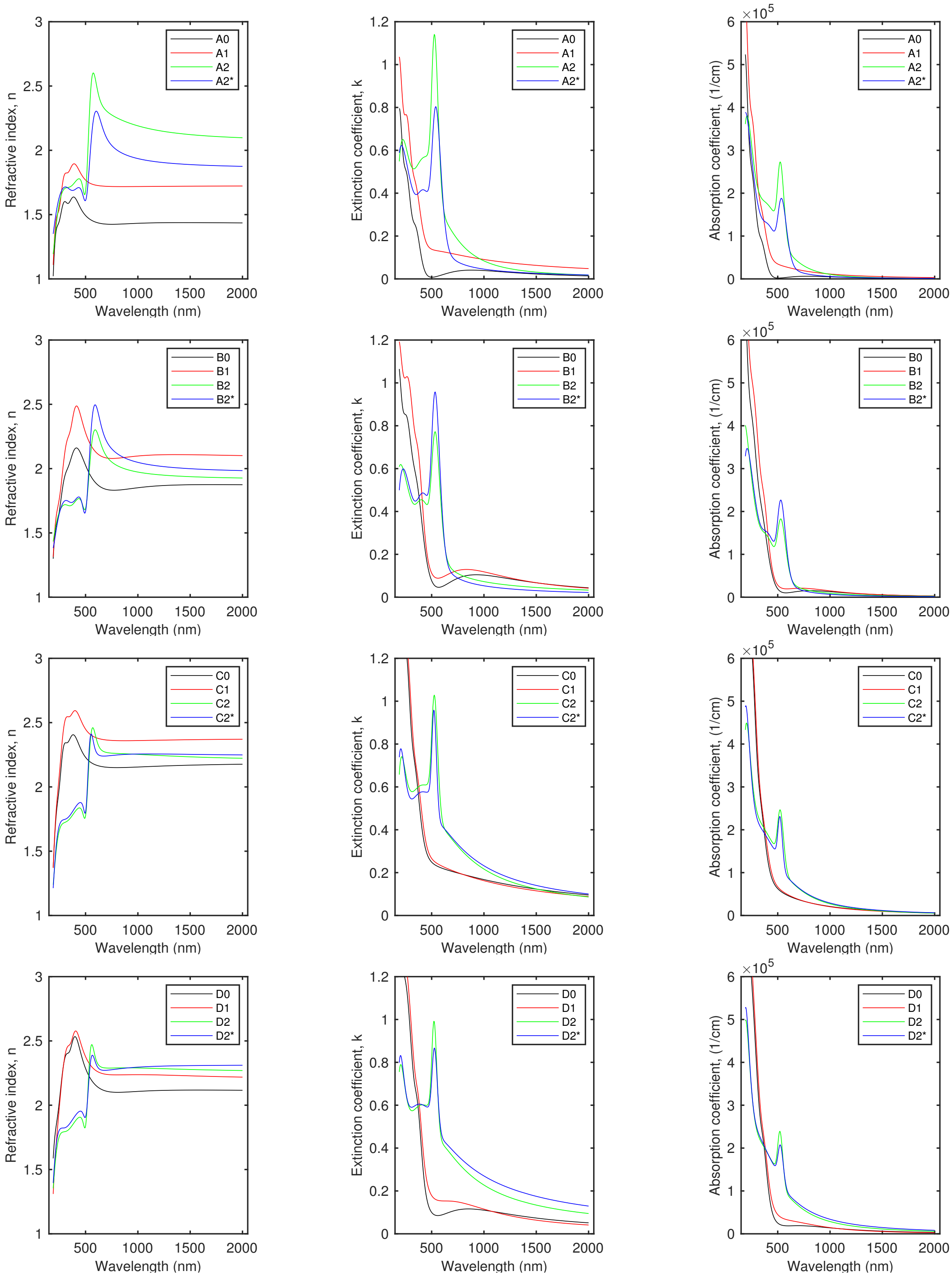

Figure S1: Refractive index (n), extinction coefficient (k) and absorption coefficient (α) determined for all the investigated samples.

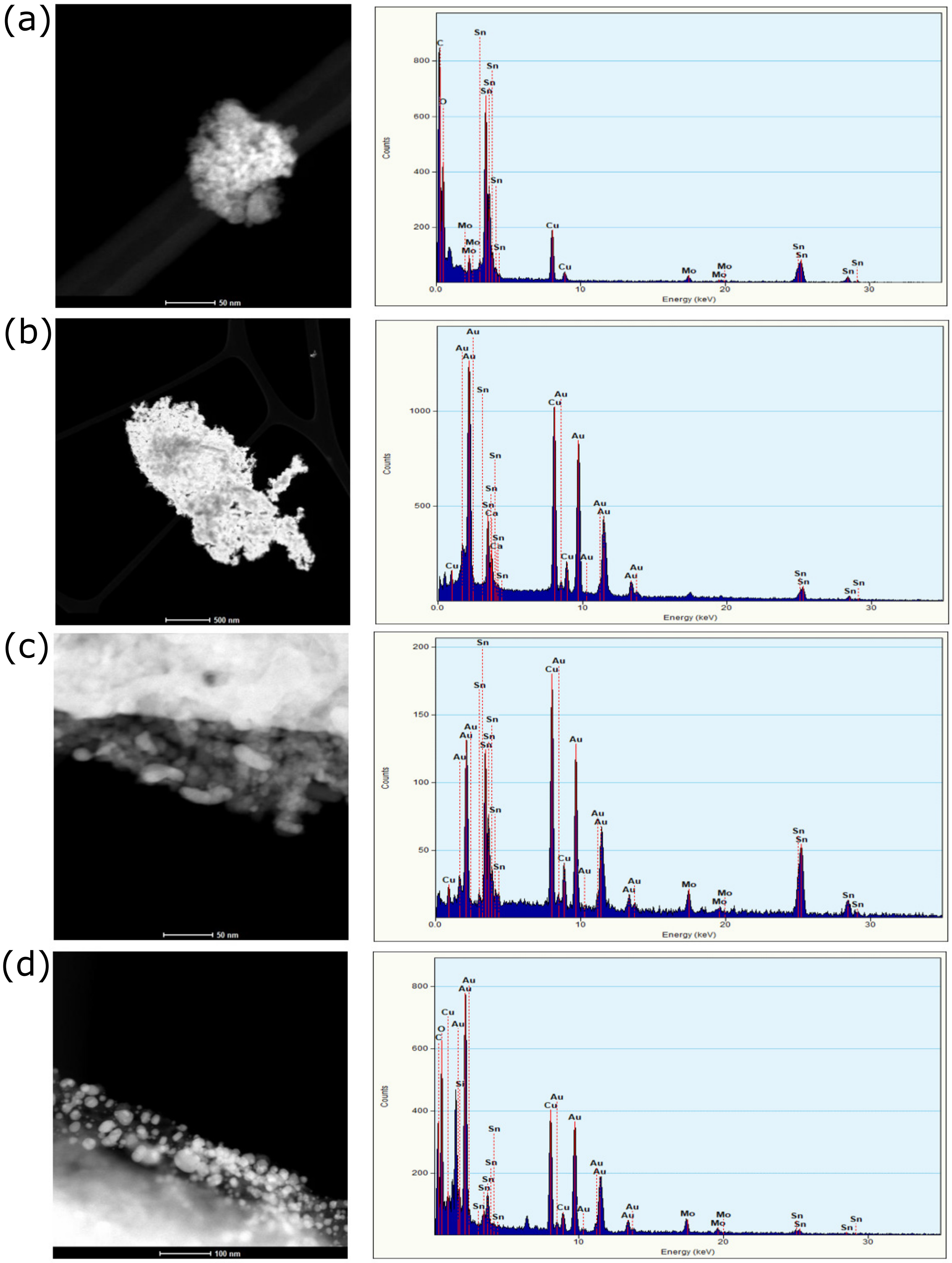

Figure S2: Energy dispersive X-ray spectroscopy analysis (EDX spectrum and corresponding STEM-HAADF image): (a) sample A2; (b) sample A2* (low magnification image including area presented in Fig. 6 (a), (e)); (b) sample B2*; (b) sample C2 (low magnification image including area presented in Fig.7 (c)). The registered Mo and Cu signals derive from the TEM instrument holder and the grid, respectively.

Table S1: Results of the EDX analysis of the samples.

Sample	Content of element (atomic %)			
	Au	Sn	Si	O
	11.39	0.58	72.11	15.90
A2	0.197	3.36	0.12	26.31
	0.01	35.87	0.27	63.83
A2*	75.24	23.03	-	1.71
	71.95	-	21.44	6.60
B2	64.93	-	5.40	29.65
	21.70	-	26.62	51.66
B2*	41.69	57.03	0.54	0.72
	25.04	46.51	9.65	18.78
C2	36.37	5.84	1.06	58.31
	19.93	2.38	5.49	72.18
C2*	100	-	-	-
	96.65	3.33	-	-
D2	22.80	-	9.07	68.12
	84.89	2.98	3.65	8.46
D2*	92.63	6.18	1.17	-
	97.27	-	2.2	-
	93.32	-	5.18	1.48

* The other elements (e.g. Mo, Cu, C) were omitted for clarity and to obtain only Au/Sn/Si/O ratio.

Table S2: Indexed Lattice Planes from the SAED Pattern presented in Figure 6f.

Diffraction No.	Indexed Diffraction Distance [nm]	Phase; Lattice Plane
1	3.369	SnO_2; (110)
2	2.637	SnO_2; (101)
3	2.336	Au; (111)/SnO_2; (200)
4	2.035	Au; (200)
5	1.760	SnO_2; (211)
6	1.587	SnO_2; (002)
7	1.433	SnO_2; (112)/ Au; (220)
8	1.210	SnO_2; (321)
9	1.093	SnO_2; (312)

Table S3: Indexed Lattice Planes from the SAED Pattern registered for sample C2.

Diffraction No.	Indexed Diffraction Distance [nm]	Phase; Lattice Plane
1	2.332	Au; (111)
2	2.087	Au; (200)
3	2.031	Si; (202)
4	1.560	SnO_2; (002)
5	1.185	Au; (222)
